# The woman with a swollen tongue: A rare case of an ANCA-negative localized eosinophil-rich granulomatous inflammation affecting solely the tongue

**DOI:** 10.1007/s00106-025-01662-9

**Published:** 2025-09-01

**Authors:** Fabio Munzinger, Luca Giudici, Michael B. Soyka, Tobias Kleinjung, Daniel Runggaldier

**Affiliations:** 1 Department of Otorhinolaryngology, Head and Neck Surgery, University Hospital and University of Zurich, Zurich, Switzerland; 2 Department of Pathology, University Hospital Zurich, Zurich, Switzerland

**Keywords:** Eosinophilic granulomatosis with polyangiitis (EGPA), ANCA-associated vasculitis, Tongue swelling, Dysphagia, Rituximab, Eosinophile Granulomatose mit Polyangiitis, ANCA assoziierte Vaskulitis, Zungenschwellung, Dysphagie, Rituximab

## Abstract

Eosinophilic granulomatosis with polyangiitis (EGPA, previously known as Churg–Strauss syndrome) is an antineutrophil cytoplasmic antibody (ANCA)-associated vasculitis (AAV) often presenting with chronic rhinosinusitis, pulmonary infiltrates, gastrointestinal and cardiac disorders, or renal lesions. Sinonasal and inner ear manifestations are common, but other affections of the head and neck area are rarely reported. Here we report a case of a young woman with a histopathological diagnosis of eosinophil-rich granulomatous inflammation affecting solely the tongue without other local or systemic lesions. It suggests histopathologically a localized EGPA according to the 1992 Chapel Hill classification but does not formally meet the recent 2022 joint classification criteria for EGPA of the American College of Rheumatology (ACR) and the European Alliance of Associations for Rheumatology (EULAR). In this report, we also describe the difficulty associated with the treatment.

## Abstract (de)

Die eosinophile Granulomatose mit Polyangiitis (EGPA, früher bekannt als Churg-Strauss-Syndrom) ist eine mit antineutrophilen zytoplasmatischen Antikörpern (ANCA-)assoziierte Vaskulitis (AAV), die oft mit chronischer Rhinosinusitis, Asthma sowie Lungeninfiltraten, gastrointestinalen, kardialen, renalen und dermalen Veränderungen einhergeht. Außerdem sind Manifestationen in den Nasennebenhöhlen und am Ohr im Krankheitsverlauf häufig, während andere Erkrankungen des Kopf-Hals-Bereichs bisher selten beschrieben wurden. In der vorliegenden Arbeit berichten wir über den Fall einer jungen Frau mit der histopathologischen Diagnose einer eosinophilenreichen granulomatösen Entzündung, die ausschließlich die Zunge betrifft – ohne nachweisbare andere lokale oder systemische Läsionen. Histopathologisch wäre die Diagnose einer lokalisierten EGPA gemäß der Chapel-Hill-Klassifikation von 1992 erfüllt. Die aktuellen Klassifikationskriterien für EGPA des American College of Rheumatology (ACR) und der European Alliance of Associations for Rheumatology (EULAR) aus dem Jahr 2022 sind hingegen nicht gegeben. In diesem Bericht gehen wir auch auf die Schwierigkeiten ein, die mit der Therapie einer solchen ANCA-negativen lokalisierten eosinophilen Entzündung verbunden sind.

## Case report

### History

**Fig. 1:**
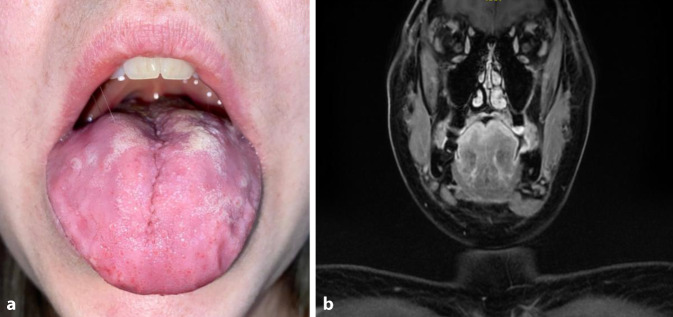
**a** Physical examination with pronounced swelling of the tongue at the initial presentation. **b** T1-weighted magnetic resonance imaging (MRI) with gadolinium: Coronal section showing strong contrast enhancement of the upper third of the tongue including the tongue surface

A woman in her 30s with a 1-week history of progressive and painful tongue swelling presented to our otorhinolaryngological (ENT) emergency department (Fig. 1a). Due to pain and burning sensations, swallowing and ingestion of solid food were restricted. There was a known allergy to pollen, dust mites, and nickel. Furthermore, bronchial asthma and recurrent rhinosinusitis, which had been self-limiting, were reported several years earlier. In that regard, no complaints were documented at the time of the patient’s presentation. The patient gave birth to a healthy child around 7 months before the onset of the tongue swelling and was otherwise healthy without regular intake of medication.

### Findings

**Fig. 2:**
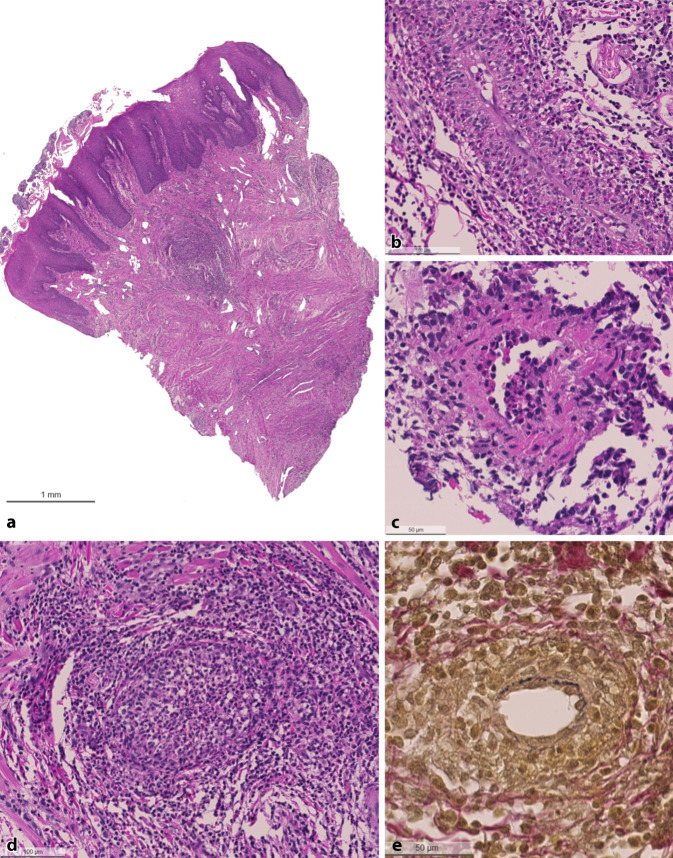
Histopathologic analysis of tongue biopsy. **a** Squamous mucosa with subepithelial eosinophil-rich granulomatous inflammation (Hematoxylin and eosin stain [H&E]). **b** Vasculitis of a small artery showing transmural eosinophil infiltration (H&E). **c,** **d** Blood vessel with eosinophil-rich inflammation and necrosis (H&E).** e** Destruction of the elastic fibers (elastic fibers are represented by black lines). Step sections. Elastica-van-Gieson, Alcian blue-PAS and Grocott (no fungi), Acid Fuchsin Orange G, Congo red (no amyloid), Ziehl–Neelsen (no acid-fast rods), Giemsa. Immunohistochemistry: kappa/lambda (polytypic plasma cells), IgG and IgG4 (physiological ratio). Spirochetes (negative)

During the clinical examination, pronounced swelling of the tongue with reduced mobility and bilaterally enlarged cervical lymph nodes were observed. The remaining clinical investigations were unremarkable; in particular, there were no clinical signs of sinusitis. The patient’s aural temperature was 36.8 °C. In the blood examination, C‑reactive protein (CRP) was slightly elevated at 6.4 mg/L and with a normal leukocyte count of 7.11 × 10^9^/L. Due to the unresponsiveness of the glossitis to low-dose systemic corticosteroids and empirical antifungal therapy, further work-up was performed with an MRI scan including contrast agent, which revealed a diffuse inflammatory process of the tongue (Fig. 1b). Next, tongue biopsies were taken, which showed eosinophilic and granulomatous inflammation with transmural vasculitis and fibrinoid necrosis of small- to medium-sized vessels. The inflammation involved the tunica intima (endothelium) first, with secondary involvement of the tunica media. There were no signs of amyloidosis, immunoglobulin G subclass 4 (IgG4) presence, or mycobacteria or fungus infestation (Fig. 2). Further diagnostic and laboratory work was provided by the Department of Immunology (Table 1). In the laboratory work-up, there was no elevated eosinophilic blood cell count. The antineutrophil cytoplasmic antibody (ANCA) status was negative. Due to myalgia of the lower extremities and enhanced metabolic activity of the calf muscles observed on positron emission tomography (PET) as well as on contrast-enhanced MRI of the gluteal, thigh, and calf muscles, muscle biopsies of the vastus medialis were taken. The histopathology of these biopsies was normal with no indications of myositis or other pathological results. The origin of the radiological changes seen in the leg muscles remains unclear.

**Table 1:** Overview of diagnostic investigations

Tests	Results
Serological	No elevation of eosinophilic granulocytes
	Normal findings of: cANCA, pANCA, ANA, ANA cytoplasm, Anti-SS‑A, Anti-SS‑B, Anti-SmD, rheumatoid factor, Anti-CCP, IgA, IgE, IgG, IgM, C1 Inhibitor, complementary factor C3c and C4, Anti-Ku 70 and 80, Anti-Mi2, IL‑1, sIL‑2, IL‑5, IL‑6, IL-12, IFN gamma, TNF alpha, free lambda and kappa light chains, HIV, Anti-HBc IgG and IgM, Anti-HBs, Anti-HCV IgG
Microbiological	Mycobacterium-specific interferon gamma release assay (QuantiFERON ®): negative
	Oral cavity swab: normal flora and *Staphylococcus aureus* with resistance to penicillin and ampicillin
Genetic	HLA-B27 and HLA-B51: negative
Cardio-/pneumological	Electrocardiogram: normal
	Echocardiography: normal
	6‑minute walking test: normal
	Lung function: normal
Radiology	MRI neck: contrast agent enhancing upper third of the tongue
	MRI thorax, abdomen, and lower extremities: symmetric contrast agent enrichment in the gluteal, thigh, and calf muscles
	PET-CT full-body scan: increased metabolic activity of the calf muscles. No indications for lymphoma or other pathologies. No chronic rhinosinusitis
Histology	Biopsies of tongue tissue: eosinophilic granulomatous inflammation with necrotic vasculitis. No indication of carcinoma, lymphoma, sarcoidosis, amyloidosis, or bacterial infection
	Biopsy of vastus medialis muscle with peripheral nerve: normal tissue without pathology

### Diagnosis

On the basis of the aforementioned findings, the diagnosis of an isolated AAV was made. Due to the histopathological characteristics of an eosinophilic and granulomatous inflammation of the tongue with necrosis of small and medium-sized vessels, and referring to the Chapel Hill Consensus Conference criteria, we suspected a pauci-immune, ANCA-negative, localized EPGA. According to the new American College of Rheumatology (ACR) and the European Alliance of Associations for Rheumatology classification, the criteria for EGPA were no longer met in this case.

### Therapy and follow-up

**Fig. 3:**
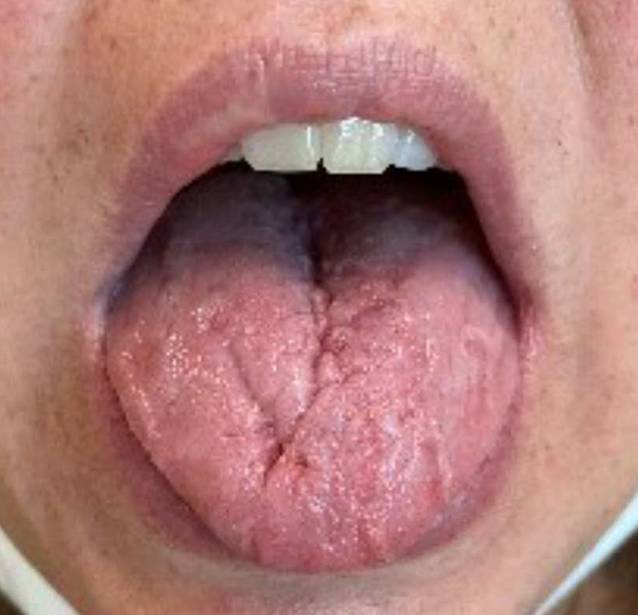
Follow-up image around 2 years after the initial treatment

The breastfeeding patient showed some relief of her symptoms after high-dose intravenous steroid therapy with 500 mg of methylprednisolone administered daily (Solu-Medrol®) and with accompanying analgesia comprising paracetamol and nonsteroidal anti-inflammatory drugs. After successive steroid reduction, the patient again complained of progressive tongue swelling and muscle pain. Treatment with the anti-interleukin-5-receptor monoclonal antibody benralizumab (Fasenra® 30 mg s.c.) and mycophenolate mofetil (CellCept®) was not successful. A combination of anti-CD20 monoclonal antibody treatment with rituximab (Mabthera®) and CellCept® was implemented. With this treatment, remission and reduction of the oral prednisone dose were achieved (Fig. 3).

## Discussion

Anti-neutrophil cytoplasmatic antibody (ANCA)-associated vasculitis (AAV) constitutes a group of systemic disorders primarily affecting small vessels [1, 2]. The AAVs can be subdivided into three entities, as defined by the 1992 Chapel Hill Consensus Conference: granulomatosis with polyangiitis (GPA, previously known as Wegener’s disease), microscopic polyangiitis (MPO), and eosinophilic granulomatosis with polyangiitis (EGPA, previously known as Churg–Strauss syndrome). The incidence of EGPA is considerably lower than other forms of AAVs with 0.5–4 cases/100,000 per year; it often presents with chronic rhinosinusitis, hearing loss, pulmonary infiltrates, gastrointestinal and cardiac disorders, renal lesions, and neuropathy [5]. Ear, nose, and throat manifestations are common; however, other affections of the head and neck area are rarely reported [5–7]. In the past, EGPA was histopathologically defined as an eosinophilic and granulomatous inflammation with necrotizing vasculitis of small and medium-sized vessels [1–3]. In 2022, the ACR and EULAR jointly proposed new classification criteria for AAV [4]. In summary, these ACR/EULAR classification criteria include clinical, laboratory, histological, and radiological parameters. In the present case with histopathological findings of eosinophilic granulomatous inflammation with necrotic vasculitis, we hypothesized a localized form of ANCA-negative EGPA or an early stage of EGPA that was still localized in one organ according to the 2012 Chapel Hill definition. However, the ACR/EULAR cut-off value for a positive EGPA classification was not met in our case [4]. In our opinion, one shortcoming of the recent ACR/EULAR classification of AAVs has become apparent in our case report of an isolated form of eosinophilic granulomatous inflammation: Localized forms of AAV as well as early stages of EGPA, which might initially present as localized and only develop into a systemic presentation at a later stage, may not be classified as EGPA based on the aforementioned ACR/EULAR classification criteria.

## Practical conclusion

- Here, we report a rare case of isolated eosinophil-rich granulomatous inflammation affecting solely the tongue.
- Discordance in the diagnosis between the 2012 Chapel Hill and the 2022 ACR/EULAR classification criteria was observed, especially in localized forms of antineutrophil cytoplasmic antibody (ANCA)-associated vasculitis (AAV) as well as early stages of eosinophilic granulomatosis with polyangiitis, which may start locally and develop into a systemic presentation later.
- In cases of unclear and persistent tongue swelling, biopsies might be helpful to diagnose autoimmune diseases such as AAVs including rare subcategories—especially if other local and systemic symptoms or lesions are absent.
- Treatment of ANCA-negative, localized eosinophil-rich granulomatous inflammation might be challenging and prolonged even when applying new therapies such as B‑cell depletion with rituximab or blocking of the interleukin (IL)-5 pathway.

## Data Availability

Previously reported studies were used to support this case report and are available on PubMed and/or Google Scholar. These datasets are cited as references at relevant places in the text [1–10].

## Declarations

For this article no studies with human participants or animals were performed by any of the authors. All studies mentioned were in accordance with the ethical standards indicated in each case. For images or other information in the manuscript that identifies patients, consent was obtained from them and/or their legal guardians.
